# Nostrum Quackery

**Published:** 1873-08

**Authors:** 


### NOSTRUM QUACKERY

Has been so often the theme of essay and dissertation, we can scarcely hope to say anything new upon the subject, and yet it is of so much interest to the profession, and so nearly touches our daily occupation, that we may venture to throw out a few random thoughts for what they may be worth.

When we walk into our drug-stores and glance over their shelving, we are confronted everywhere with an army of nostrums which are evidently displayed for a purpose—they certainly *mean business*. If we should possess so far the confidence of the proprietors as to be permitted to inspect the ledger, we would probably find that the sales of these so-called remedies yield a large per centage of the profits of the business.

It is useless to blink the fact that these preparations, whether for weal or for woe, supply a great public want; *the people will have them*, in spite of all we may do or say.

It is notoriously true that all efforts thus far made by the medical profession to suppress the great and growing evil have most signally failed. Why is this?

There are many reasons which might be assigned, but for the present we shall confine our attention to two: 1. Nostrums supply a want which the community feel, and for which they are willing to pay their money; 2. The opposition of the medical profession to the sale of quack preparations is set down to selfish greed, and has not yet been divested of the *appearance*, at least, of self-seeking.

Until we of the profession are willing to place ourselves in a position of pecuniary disinterestedness, and, at the same time, to acknowledge and supply the want of the public for household remedies, we may as well 'bate our breath in the matter.

It is fairly objected to the present system of nostrum quackery that it is dangerous to the health and lives of the people, because the composition in constituents and quantities of its preparations is withheld from the profession and the public. If alarming symptoms ensue, there is no means of determining the agency of the medicine in the case. If serious illness follows, the attending physician is left in the dark as to the medicines which have actually been taken in the compound, and their probable influence upon the symptoms present.

These remedies are dangerous, because of the difficulty of effecting perfect combination of materials when operating upon a large scale, as witness the case of poisoning by "Mrs. Winslow's Soothing Syrup," at San Francisco and at St. Louis. They are dangerous, because of the liability to error incurred in the employment of cheap and unskilled labor for many of the details of manufacture in very large establishments.

They are objectionable, because there is no recourse to be had upon the manufacturer in a distant State, in the event of death or injury resulting.

In the way of *remedy* for these acknowledged evils, we suggest that the medical profession recognize the want of household remedies by publishing *authoritative formulæ* for the use of pharmacists, who may compound them at their counters and keep them on hand for the ready and convenient supply of *all the real wants* of families and of individuals in this direction.

Let vendors of patent and proprietary medicines and nostrums be required by law to fully and distinctly set forth upon their labels the names and quantities employed of all the materials used in the compound, together with a working formula; and let suitable penalties be attached to all fraud and deception with reference to the compound.

Moreover, let physicians encourage the establishment of small pharmacies in skilled hands, from which all nostrums which do not conform to the above requisitions are rigidly excluded; and when this is impracticable, let them attach dispensing apartments to their own offices. B.

